# Cost-effectiveness of an HPV self-collection campaign in Uganda: comparing models for delivery of cervical cancer screening in a low-income setting

**DOI:** 10.1093/heapol/czx076

**Published:** 2017-08-23

**Authors:** Nicole G Campos, Vivien Tsu, Jose Jeronimo, Denise Njama-Meya, Mercy Mvundura, Jane J Kim

*Health Policy and Planning,* doi: 10.1093/heapol/czw182. The following conflict of interest statement was missing: Dr Jose Jeronimo was the co-owner and Deputy Manager of Onco Prev International, a Peruvian company, from 2012 through March 2017. Onco Prev offers cervical cancer screening services and in 2016 also began positioning for distribution of medical devices including colposcopes and the Liger thermocoagulator. Onco Prev International did not commercialize any medical instrument during the time Dr Jeronimo was part of the company.

